# Cow-Pock Institution

**Published:** 1808-05

**Authors:** J. S. Knowles

**Affiliations:** Resident Inoculator. Central House


### *To the Editors of the Medical and Physical Journal.*

GENTLEMEN,

I Have the honour to forward, for insertion in your Journal, the Report of the Dublin Cow-Pock Institution for the year 1807, transmitted to the Royal Jennerian Society.

I am, &c.

J. S. KNOWLES,
Resident Inoculator.

*Central House, April* 23, 1808.

---

### COW-POCK INSTITUTION.

Under the Patronage of his Grace the Lord Lieutenant.
Removed from North Cope-Street, to No. 55, Sackville-Street.

*Opened on the* 14*th of January,* 1804, *under the direction of the undersigned Physicians and Surgeons of this City, for the purposes of securing a succession of Cow-Pock Matter, of inoculating gratuitously the Children of the Poor, and of supplying the different parts of the Kingdom with genuine Infection.*

| *Physicians.* | *Surgeons.* |
|---|---|
| JOSEPH CLARKE, | GEORGE STEWART, |
| JAMES CLEGHORN, | RALPH S. OBRE, |
| THOMAS EVORY, | SOLOMON RICHARDS. |

*An Abstract from the Register of Inoculations and Distribution of Matter.*

| | Patients inoculated. | Packets issued to Practitioners in general. | Packets to Army Surgeons. |
|---|---|---|---|
| 1804 | 578 | 776 | 236 |
| 1805 | 1,032 | 1,124 | 178 |
| 1806 | 1,356 | 1,340 | 220 |
| 1807 | 2,156 | 1,790 | 320 |
| Total | 5,122 | 5,030 | 954 |

The Directors of the Cow-Pock Institution, have great pleasure in offering to the public, a fourth annual Statement, which agrees with their former Reports, in establishing the safety and efficacy of vaccine inoculation.—They are happy to state, that no case has occurred to them of smallsmall-pox succeeding perfect vaccination. They have additional satisfaction in observing, that the accounts of failure are less numerous within the last year than formerly; and they hope the period is not distant, when practitioners shall have acquired such a knowledge of the new inoculation as will effectually prevent such occurrences. A few cases have been mentioned, where small-pox attacked children who had been vaccinated at the Institution; there was no certainty, however, of their having regularly gone through the cow-pock, as they did not attend at the proper periods after being inoculated.

The strictest attention has been paid to investigate the influence of cow-pock upon the constitution, and in no instance has it appeared to have occasioned any permanent injury. The mothers of many children who have been inoculated during the present year, had others also inoculated at different times since the opening of the institution. These parents, satisfied of the efficacy of cow-pock in protecting their children against small-pox, bring forward no cases of eruption or other complaints following vaccination.

The success of the new inoculation in lessening the frequency of small-pox has of late been very evident in this city. Some practitioners, who, in compliance with vulgar prejudices, still practise variolous inoculation, frequently complain of the difficulty of procuring infection. Similar complaints are made by medical people in some country towns, whence they have been known to send to Dublin for small-pox matter. A town is mentioned by a correspondent, in which the small-pox has for some time been unknown, until lately introduced by an itinerant inoculator.

Several instances of the co-existence of small-pox and cow-pock have occurred within the present year, in none of which did the latter appear to have had any influence in lessening the severity of the former.

In November the cow-pock, in a greater number of children than usual, assumed an irregular appearance without any apparent cause. In some the disease preserved its characteristic marks until the seventh or eighth day, after which the vesicle was suddenly converted into a sore, disposed to form purulent crusts, sometimes attended with a considerable erysipelatous inflammation. If the *scabbing process* had not been particularly attended to, this irregularity might have been overlooked, which points out the necessity of attending to the disease, through all its stages. In others, the vesicle, from its commencement, was decidedly spurious, *globular* in its form, contents *not limpid, ea-*

*sily ruptured,* surrounded by a premature areola, and, much within the usual time, formed a dark mahogany scab, which preserved the globular form of the vesicle. In two cases irregular vesications surrounded the vaccine vesicle, which becoming confluent, terminated in troublesome ulceration, which destroyed the vaccine vesicle.—In one, where the arm had a perfectly regular appearance on the eighth day, the areola extended over the arm and forearm, the side, and lower extremity of the side inoculated, accompanied with considerable hardness, fever, &c.; in a few days however the inflammation subsided and the child got well.—Some of the children on whom these anomalies appeared were a second time vaccinated, and the disease ran a similar course.

A remarkable instance of the suspension of the vaccine virus was observed.—A child was twice inoculated, apparently without effect—at the expiration of eight months the operation was repeated in two parts of the arm—both incisions inflamed and vesicated naturally. One of the cuts from the former inoculation began to inflame on the fourth day from the third operation, and ran a regular course, the vesicle, areola, and scabbing process being perfectly natural. The recently produced vesicles were accidentally rubbed off, and ran on to ulceration.

The increased experience of the institution tends to confirm the opinion respecting the influence of herpetic and other eruptions on the skin in modifying the vaccine disease. It is at present the usage of the institution to decline vaccination under such circumstances, until the complaint of the skin be apparently removed.

Several children, labouring under hooping cough, rickets, scrofula, and some other chronic diseases, have been vaccinated, in none of whom was there any thing peculiar remarked.—The supervening of chicken pock has in a few deranged the vaccine disease.

In cases where the vesicle from neglect has been much rubbed, or otherwise injured, ulceration on the arm, and abscess in the axilla have occasionally occurred.

By the extended correspondence of the institution, it appears that the cow-pock has made great progress throughout Ireland within the present year.—The clergy and country gentlemen have in many instances been very zealous in co-operating with medical practitioners, and in some counties, societies have been formed for the gratuitous inoculation of the poor. Its general adoption also in the army, has had a great effect in extending the practice.

SAMUEL B. LABATT, Secretary.

*Dublin, Dec.* 31, 1807.

